# Analysis of the Polyphenolic Composition of *Vaccinium* L. Extracts and Their Protective Effect on Red Blood Cell Membranes

**DOI:** 10.3390/membranes13060589

**Published:** 2023-06-07

**Authors:** Teresa Kaźmierczak, Dorota Bonarska-Kujawa, Katarzyna Męczarska, Sylwia Cyboran-Mikołajczyk, Jan Oszmiański, Ireneusz Kapusta

**Affiliations:** 1 Department of Biotechnology, University of Wrocław, Joliot-Curie 14a, 50-383 Wrocław, Poland; 2 Department of Physics and Biophysics, Wrocław University of Environmental and Life Sciences, Norwida 25, 50-375 Wrocław, Polandkatarzyna.meczarska@upwr.edu.pl (K.M.); sylwia.cyboran@upwr.edu.pl (S.C.-M.); 3 Department of Fruit, Vegetable and Plant Nutraceutical Technology, Wrocław University of Environmental and Life Sciences, Chełmońskiego 37, 51-630 Wrocław, Poland; jan.oszmianski@upwr.edu.pl; 4 Institute of Food Technology and Nutrition, University of Rzeszów, Zelwerowicza 4, 35-601 Rzeszów, Poland; ikapusta@ur.edu.pl

**Keywords:** plant extract, blueberry polyphenols, erythrocytes, lipid membrane, liposomes, HPLC, fluorimetry

## Abstract

The blueberry fruit of the genus *Vaccinium*, including high blueberry, low blueberry, and wild bilberry, is consumed for its flavor and medicinal properties. The purpose of the experiments was to investigate the protective effect and mechanism of the interaction of blueberry fruit polyphenol extracts with the erythrocytes and their membranes. The content of polyphenolic compounds in the extracts was determined using the chromatographic UPLC–ESI–MS method. The effects of the extracts on red blood cell shape changes, hemolysis and osmotic resistance were examined. Changes in the order of packing and fluidity of the erythrocyte membrane and the lipid membrane model caused by the extracts were identified using fluorimetric methods. Erythrocyte membrane oxidation was induced by two agents: AAPH compound and UVC radiation. The results show that the tested extracts are a rich source of low molecular weight polyphenols that bind to the polar groups of the erythrocyte membrane, changing the properties of its hydrophilic area. However, they practically do not penetrate the hydrophobic part of the membrane and do not damage its structure. Research results suggest that the components of the extracts can defend the organism against oxidative stress if they are delivered to the organism in the form of dietary supplements.

## 1. Introduction

The blueberry fruits of the genus *Vaccinium* consist of highbush blueberries HB (*Vaccinium corymbosum* L.), lowbush blueberries LB (*Vaccinium angustifolium* L.) and European blueberries, also called wild bilberries WB (*Vaccinium myrtillus* L.), are of great commercial importance and mainly consumed in processed products and food supplements. Phytochemical isolates from WB and cultivated HB and LB have beneficial effects in preventing diseases attributed to free radicals’ production [1,2]. The interest in these species is due to their high content of phenolic compounds, which are secondary plant metabolites. In addition, they are well-known for their health protection attributes as anti-inflammatory, antihypertensive, antimicrobial, and anticancer agents [3,4,5]. Furthermore, blueberries can inhibit colon, cervix, prostate, liver, and triple-negative breast cancer growth [3,6,7,8,9,10,11,12,13,14,15,16,17].

Moreover, blueberries have been shown to improve insulin sensitivity, reduce the risk of type 2 diabetes, and have protective effects against Alzheimer’s disease, memory decline, and neuro- and mitochondria-destruction. In addition, blueberries provide cardiovascular benefits like reduced cholesterol, lower blood pressure, help related to metabolic syndrome, protection against stroke and reduction of oxidative stress [3,7,8,18,19,20,21,22,23]. Furthermore, the administration of polyphenols such as catechins, anthocyanins, and procyanidin, the main components of blueberry fruits, stimulated the growth of various probiotic bacteria. This leads to a reduction in fat mass, regulation of glucose metabolism, reduction in fatty liver and weight loss [24].

Scientists are studying how natural compounds or phytonutrients in blueberries may help combat diseases, including lifestyle diseases, largely caused by oxidative stress. In many physiological processes or as a result of external physicochemical factors, free radicals and their accumulation contribute to, among other, the oxidation of cell membranes. Thus, it can lead to pathological states in the organism. Lipid oxidation by free radicals changes the physicochemical properties of the cell’s lipid membrane, e.g., by reducing the hydrophobicity inside the lipid membrane, changing the organization and fluidity of the membrane, lipid asymmetries, and membrane depolarization [25,26,27]. As a result of these changes, the activities of enzymes and transport proteins are inhibited, and membrane integrity can be compromised, leading to cell death. Today, efforts to find new effective substances to help prevent and treat some dangerous diseases are very important. In addition to studies showing the health benefits of plant extracts and polyphenol compounds, it is possible to use these substances only for appropriate protection and treatment and understand the mechanism of their interactions with living cells. They have antioxidant and anti-inflammatory effects and are helpful in the treatment of tumors, nerve, digestive, bone, teeth, urinary and blood diseases [17,23,28,29,30,31]. In biophysical scientific methods, we can observe the interaction of these compounds with cell structures, especially with biological membranes. In contact with a living organism, any biologically active substance first reacts with the cell membranes. This is often the only way such substances interact with living organisms. Understanding the mechanisms of the molecular effects of polyphenol compounds on organisms is of critical importance from the point of view of medicine, but also pharmaceutical, food, and other related technologies. It should be noted that the mechanisms of interaction between individual polyphenol compounds and plant extracts with biological membranes are not yet fully understood, so the authors have undertaken research in this area.

## 2. Materials and Methods

### 2.1. Materials, Reagents and Standards

Chlorogenic acid and glycolyzed derivatives of cyanidin, delphinidin, malvidin, petunidin, peonidin and quercetin were purchased from Extrasynthese (Lyon, France). The fluorescent probes 6-dodecanoyl-2-dimethylaminonaphthalene (Laurdan) and 1,6-diphenyl-1,3,5-hexatriene (DPH) were purchased from Molecular Probes (Eugene, OR, USA). Acetonitrile was purchased from Merck (Darmstadt, Germany). Formic acid, methanol, and oxidation inductor 2,2′-azobis (2-methylpropionamidine) dihydrochloride (AAPH) were purchased from Sigma Aldrich (Steinheim, Germany).

### 2.2. Plant Material

Highbush blueberries (HB) (*Vaccinium corymbosum* L.) were obtained from the commercial plantation of Szczecin (Poland). Lowbush blueberries (LB) (*Vaccinium angustifolium* L.) were collected from the Rajkowo Experimental Station near Szczecin in Poland. Wild bilberry fruit (WB) (*Vaccinium myrtillus* L.) was harvested in a forest near Szczecin in Poland. The extracts of plants were prepared in the Department of Fruit, Vegetable and Plant Nutraceutical Technology of Wroclaw University of Environmental and Life Sciences.

### 2.3. Red Blood Cells

The hemolytic and microscopic studies were conducted on fresh, heparinized pig blood. The pig blood used was acquired from the slaughterhouse and was purchased commercially, which under Polish law, does not require the consent of the bioethics committee. The selection of pig erythrocytes was determined by their cells’ lipid composition being the most similar to human erythrocytes [32], and blood was easily available. Erythrocytes were prepared according to the procedure described by Cyboran-Mikołajczyk [32].

### 2.4. Erythrocyte Membranes

Fluorescence studies were conducted on isolated erythrocyte membranes (ghosts) from fresh blood using the Dodge method [33]. Erythrocyte membranes were suspended in an isotonic phosphate solution of pH 7.4 in such quantity that the protein concentration in the samples was approximately 100 mg/mL. Membrane protein concentration was assayed using Bradford’s method [34].

### 2.5. Lipid Membranes

The lipids of red blood cells (RBCLs) were extracted from the erythrocyte membranes according to the methodology described by Maddy [35]. The final lipid concentration was 10 mg/mL. The resulting lipid solution was stored in the freezer at −30 °C. Small unilamellar liposomes (SUV) for fluorimetric experiments were made according to the procedure described by Bonarska-Kujawa [36].

### 2.6. Extracts and HPLC–DAD and UPLC–ESI–MS Analysis

Polyphenols were isolated from fruits by extraction with water containing 200 ppm of SO_2_, the ratio of solvent to fruits being 3:1. The extract was absorbed on Purolite AP 400 (Llantrisant, Wales, UK) for further purification. The polyphenols were then eluted with 80% ethanol, concentrated and freeze-dried.

The polyphenols extraction procedure was described by Gąsiorowski et al. [37]. The extracts’ percent content of low molecular weight polyphenols was determined using high-performance and ultra-performance liquid chromatography (HPLC and UPLC). Flavonoids and phenolic acids were identified with the HPLC–DAD (High-Performance Liquid Chromatography with Diode-Array Detection) method and the UPLC–ESI–MS (Ultra-Performance Liquid Chromatography-Electrospray-Tandem Mass Spectrometry) analysis described extensively by Oszmiański [38,39] and Teleszko [40].

The UPLC–ESI-MS analysis conditions were LC System Aquity UPLC Waters, (Etten-Leur, The Netherlands) column: BEH C18 2.1 × 50 mm, 1.7 μm, temperature 50 °C, flow rate 0.35 mL/min, mobile: phase A: 0.1% acetic acid in the water, phase B: 0.1% acetic acid in 40% acetonitrile, gradient: initial 80% A, 80% A–50% A in 3 min.

In MS analysis, all mass spectra were acquired using a triple quadruple mass spectrometer (Aquity TQD, Waters) equipped with an electrospray ionization source. Positive and negative-ion mass spectra were acquired. Positive and negative-ion ESI was performed using a capillary voltage of 3.0 kV, and cone voltage of 50 V. Nebulization was achieved using nitrogen gas at a flow of 800 L/h.

Desolvatation was aided using a temperature of 350 °C. Mass spectra were recorded over the range of 80–1100 *m*/*z*. Tandem mass spectra were obtained using manual MS/MS by isolating the base peak (parent ion) from the direct infusion. Samples were dissolved in MeOH and infused in the ESI source by a syringe pump at a 5 μL/min flow rate. The mass spectroscopic data were acquired and processed using MassLynx 4.1 software (Milford, CT, USA).

### 2.7. Antioxidant Activity of Extracts

The antioxidative activity of HB, LB and WB extracts was determined using the fluorimetric method described earlier by Bonarska Kujawa [41]. These studies were carried out on erythrocyte membranes. The DPH-PA probe was used in the experiments at a final concentration of 10 µM in the sample. Suspensions of erythrocyte membranes were treated with UVC radiation (3.5 mW/cm^2^) and a chemical oxidation inducer (AAPH) for 30 min. The AAPH compound was dissolved in distilled water at a concentration of 1 mM, and the final concentration in the sample was 20 µM. Free radicals, released during UVC irradiation or AAPH decomposition, cause quenching of DPH-PA fluorescence and decreased fluorescence intensity. Therefore, relative fluorescence, i.e., the ratio of UVC or AAPH-oxidized probe fluorescence to the initial fluorescence of the probe, was adopted as a measure of the extent of lipid oxidation. Excitation and emission wavelengths of the DPH-PA probe were λ_ex_ = 364 nm and λ_em_ = 430 nm.

### 2.8. Hemolytic Activity of Extracts and Osmotic Resistance of Erythrocytes

Hemolytic and osmotic resistance experiments were carried out on the fresh blood of pigs and investigated using the spectrophotometric method described by Cyboran-Mikołajczyk [42] with minor changes. The hemolytic activity of extracts was determined based on hemoglobin concentration released from erythrocytes modified with extracts for 1 h at 37 °C. For osmotic resistance, the erythrocytes modified by the extracts for 1 h at 37 °C were taken and suspended in test tubes containing NaCl solutions of 0.5–0.86% concentration and an isotonic (0.9%) NaCl solution. Using the obtained results, the NaCl percent concentrations (C_50_) that caused 50% hemolysis of erythrocytes were found. The C_50_ values were taken as a measure of osmotic resistance.

### 2.9. Erythrocyte Shapes

The shapes of erythrocytes modified with extracts from blueberry fruits of the genus *Vaccinum* were determined using a Nikon Eclipse E200 (Amstelveen, The Netherlands) optical microscope and a scanning electron microscope (EVO LS15 ZEISS, Oberkochen, Germany). The method used was previously described by Bonarska-Kujawa et al. [43]. The morphological index of the individual form of erythrocytes was given according to the Bessis scale [44], i.e., a negative value of −1 to −4 for stomatocytes and a positive value of 1 to 4 for echinocytes. For microscopic examination, 1 h modification of erythrocytes was performed at 37 °C with a 0.1 mg/mL concentration of HB, LB, and WB extracts.

### 2.10. Fluidity and Packing Arrangement of the Membranes

The effect of HB, LB and WB extracts on packing order and fluidity of erythrocyte membranes and model lipid membranes (RBCL liposomes) was studied by the fluorimetric method described by Bonarska-Kujawa [45] with minor modifications.

The fluorescent intensity was measured by the DPH and Laurdan probes at 37 °C, and their concentration in the sample was 10 mM. The extract concentration in the sample was 0.005 to 0.025 mg/mL.

The small unilamellar liposomes (SUVs) were composed of lipids extracted from the erythrocyte membranes (RBCL) and were formed by a sonicator. The control samples contained only lipid suspension with a fluorescence probe at 100:1 lipids: fluorescent molar ratio, an appropriate extract at a concentration of 0.005–0.025 mg/mL being added to the remaining samples.

Based on the measures of the fluorescence intensity of the probes, the values of fluorescence anisotropy (A) for the DPH probe and generalized polarization (GP) for the Laurdan probes were calculated using the formula according to Parasassi et al. and Lakowicz [46,47].

### 2.11. Statistical Analysis

Statistical analysis was performed using Statistica 12.0 (StatSoft Inc., Hamburg, Germany). All experiments were conducted in at least three separate batches unless otherwise indicated. The variation analysis was carried out, and the significance between the mean was determined using Dunnett’s post-hoc test. The results were presented as mean ± SD, and significant levels were defined at *p* < 0.05.

## 3. Results

### 3.1. HPLC–DAD and UPLC–ESI–MS Analysis

The extracts were analyzed using UPLC–ESI–MS and HPLC–DAD systems. The qualitative analysis obtained by the LC–MS method and quantitative analysis by the HPLC (quantification by DAD detection) is summarized in Table 1. 20 low molecular weight polyphenols in the extracts of highbush and lowbush blueberry fruit, and 18 in the extracts of wild bilberry fruit were identified and quantified. Hydroxycinnamate and chlorogenic acid were also detected. The compounds with [M-H]^−^ at *m*/*z* 353 with λ_max_ = 320 nm gave caffeic acid after fragmentation. UPLC–ESI–MS spectra are included in Supplementary Materials (Figures S1–S3).

The five categories of anthocyanins corresponding to the galactosides, glucosides, and arabinosides of delphinidin, cyanidin, petunidin, peonidin, and malvidin were identified from reference compounds using UV–VIS and mass spectra, and the literature [48].

Direct injection ESI–MS analysis was unable to differentiate anthocyanin isomers, such as cyanidin-3-glucoside/galactoside and petunidin-3-arabinoside (equivalent molecular weight of 449), peonidin-3-galactoside/glucoside and malvidin-3-arabinoside (equal molecular weight of 463). MS/MS analysis separated all anthocyanins into two groups, according to their sugar substitutions: loss of 162U for glucosides and galactosides of anthocyanin and loss of 132U for arabinosides [49]. Four quercetin derivatives were detected in highbush and lowbush blueberries: quercetin-3-O-galactoside, quercetin-3-O-glucoside, quercetin-3-O-arabinoside and quercetin-3-O-rhamnoside. The two quercetin derivatives: quercetin-3-O-galactoside, quercetin-3-O-glucoside were identified in wild berries (Table 1). All compounds had a fragmentation that yielded a quercetin-negative ion at *m*/*z* = 301 [50,51].

The anthocyanidin derivatives were the most predominant phenolic group found, constituting 25.11% in HB, 27.54% in LB, and 31.52% in WB extract, respectively. Among identified anthocyanidin derivatives, the most important compounds were cyanidin-3-O-galactoside (6.3%) in HB, delphinidin-3-O-galactoside (7.97%) in LB and delphinidin-3-O-glucoside (6.12%) in WB extract, respectively.

The other compounds, such as chlorogenic acid and quercetin derivatives, accounted for 12.72% of highbush blueberries, 16.36% of lowbush blueberries, and 2.46% of wild bilberry fruit powders.

### 3.2. Antioxidant Activity of Extracts

The antioxidant activities of HB, LB and WB extracts were studied on the erythrocyte membrane. First, the antioxidant activity was determined fluorometrically based on the kinetics of the DPH–PA fluorescence suppression caused by free radicals induced by UVC radiation and the AAPH compound [52]. In addition, the concentration of 50% inhibition of erythrocyte membranes lipids oxidation (IC_50_) was determined based on the kinetics of the oxidation curve obtained for the extracts and Trolox^®^ -treated as a standard antioxidant (Table 2).

The results showed that polyphenolic extracts protect the red blood cell membrane lipids from the free radicals-induced oxidation caused by UVC radiation and the AAPH compound. The degree of protection of the membrane lipids demonstrated by the preparations was comparable to that of Trolox^®^ for AAPH. However, it was much smaller than that determined for Trolox^®^ against free radicals induced by UVC radiation. The results showed that WB extracts protect the erythrocyte membrane from UVC oxidation much better than HB and LB extracts but worse than Trolox^®^. To protect membranes from AAPH–induced oxidation, HB and LB extracts were as good as Trolox^®^, but worse than WB extract. The results indicated that polyphenols in the tested blueberry extracts have good antioxidant properties, and they are more effective in scavenging free radicals generated by the chemical agent AAPH than UVC radiation.

### 3.3. Hemolytic Activity of Extracts and Osmotic Resistance of Erythrocytes

The presence of HB, LB and WB extracts with concentrations ranging from 0.01 to 0.1 mg/mL did not increase hemolysis of erythrocytes compared to control cells (1.54 ± 0.55%). At 0.1 mg/mL concentration of extracts, hemolysis was 1.11 ± 0.17% for WB, 2.08 ± 0.77% for HB, and for LB extract, it was at 1.97 ± 0.46%.

In the study of the extracts’ effects on erythrocyte osmotic resistance, no significant differences between control cells and those treated by the extracts with different concentrations of sodium chloride were obtained. The C_50_ values of blood cells modified with extracts of HB, LB and WB with 0.05 mg/mL concentration as follows: control—0.731 ± 0.036, HB—0.733 ± 0.037, LB—0.726 ± 0.042 and WB—0.722 ± 0.021 of NaCl [%] concentration. The results indicate that extracts from the range of concentrations used do not change erythrocyte resistance to changes in osmotic pressure.

### 3.4. Erythrocyte Shapes

**Figure 1:**
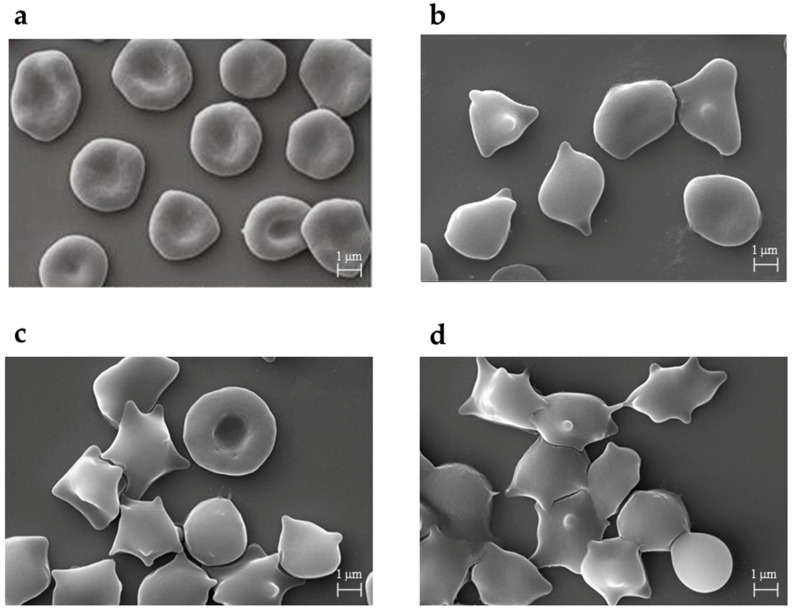
Shapes of unmodified erythrocytes (**a**), modified with high blueberry (**b**), low blueberry (**c**), and wild bilberry (**d**) were observed with an electron microscope (SEM) at 0.1 mg/mL concentration. Magnifications were 5000×.

**Figure 2:**
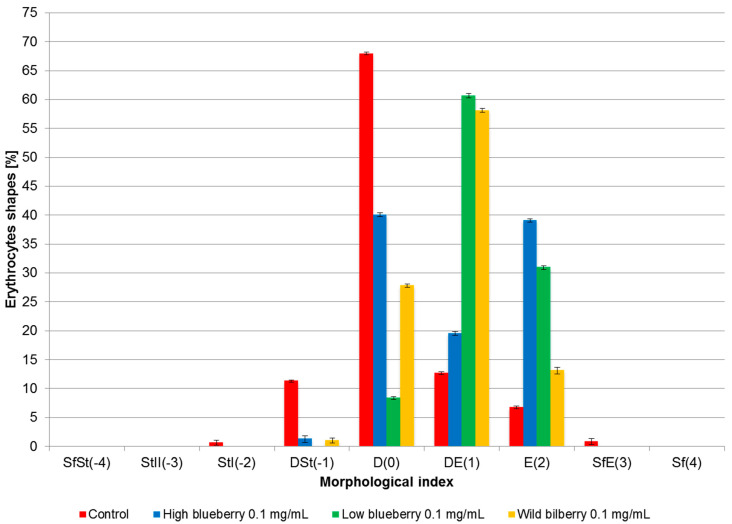
Percent share of different shapes of erythrocytes induced by blueberry extracts at concentration 0.1 mg/mL. On the abscissa, there are morphological indices for the respective shapes of cells: sphero-stomatocytes (SfSt (−4)), stomatocytes II (StII (−3)), stomatocytes I (StI (−2)), disco-stomatocytes (DSt (−1)), discocytes (D (0)), disco-echinocytes (DE (1)), echinocytes (E (2)), sphero-echinocytes (SfE (3)) and spherocytes (Sf (4)).

Figure 1a–d shows the shapes of the erythrocytes observed in the scanning electron microscope. Figure 2 shows the percentage of different cell forms of erythrocytes modified by HB, LB, and WB extracts at a concentration of 0.1 mg/mL. As shown in Figure 2, the treatment of extracts on RBCLs triggered different forms of echinocytes, especially disco-echinocytes. Studies by Deuticke [54] and Iglic [55] have shown that the formation of echinocytes occurs when amphiphilic molecules are incorporated into the outer monolayers of the erythrocyte membrane. Consequently, extracts of blueberries can be assumed to concentrate mainly on the outer layer of the erythrocyte membrane when inducing echinocytes and virtually not penetrate the inner layer of the membrane.

### 3.5. Fluidity and Packing Arrangement of the Membrane

The anisotropy of the stable DPH state is mainly related to the rotational movement restriction due to the packing order of the hydrocarbon chain. Thus, the decrease in this parameter can be explained by structural disturbances in the hydrophobic region of the bilayer caused by the incorporation of the investigated compounds. The effect of HB, LB, and WB extracts on the fluidity of erythrocyte membranes and liposomes formed from RBCLs was studied using fluorescence anisotropy (A). The results for such membranes are shown in Table 3. Used compounds practically do not change the anisotropy of the DPH probe located in the erythrocyte membrane. A small fluctuation in DPH anisotropy was observed only for the highest concentration of HB and LB extracts. No significant changes in the RBCL liposomes affected by all used extracts were observed in the hydrophobic region, where the unspecified DPH probe was located. Thus, the preparations can be postulated that it does not concentrate in the hydrophobic lipid phase of the erythrocyte membrane. The results indicate that the extracts do not alter the fluidity of the erythrocyte membrane in the region occupied by the fatty acid chain of lipid molecules [27]. The Laurdan probe was also examined for the degree of order of the hydrophilic part of the erythrocyte membrane and the liposomes formed from RBCL. The calculated general polarization values (GP) decreased as the concentration of extracts increased (Table 4), which indicates that the hydrophilic part of the erythrocyte membrane is becoming more disorganized. These changes are probably caused by the penetration of the components of the extracts into this region or through absorption on the membrane surface.

No significant extract-induced changes in the hydrophilic part of RBCL liposome membranes were found. However, changes in the erythrocyte membrane induced by the extracts caused less order (GP) in the hydrophilic part of the erythrocyte membrane.

The conviction remains that the extract components integrate into the erythrocyte surface through interactions with glycocalyx, but in RBCL membranes, extract components locate more superficially, remaining in the aqueous phase near the surface of the lipid membrane.

## 4. Discussion

The main objective of the research included in this publication was to determine the antioxidant activity and the effect of selected polyphenolic extracts from highbush, lowbush, and wild blueberries on the physical properties of biological and lipid membranes. In addition, the research was aimed at determining the likely mechanism of interaction of the low-molecular fraction of blueberry polyphenols with biological membranes based on the effects induced in them. This study treated erythrocytes as an example and model of a biological membrane and cell. Therefore, they will serve as a model system for the changes induced by polyphenolic extracts, likely to occur in most cell types.

Qualitative and quantitative analysis of the extracts of fruits from highbush, lowbush, and wild blueberries showed that they are rich in low-molecular-weight phenolic compounds. The main flavonoid components of the fruit extracts were the following anthocyanins: derivatives of delphinidin (glucoside, galactoside, arabinoside, rutinoside) and derivatives of cyanidin (galactoside, arabinoside, glucoside) and a small number of derivatives of malvidin, peonidin, petunidin. Anthocyanins predominated (above 90% of low molecular weight polyphenols) in wild bilberry extract. Low and highbush blueberry extracts (LB and HB) contained, in addition to anthocyanins, chlorogenic acid, about 30% of the identified compounds [54].

The results of the HPLC-DAD measurements showed that anthocyanins maximum light absorptions were in the 514–526 nm range. This contributes to the fact that these polyphenolic components absorb mainly green light, which contributes to red, blue, and purple light emissions. Consequently, it gives the blueberries their characteristic color. The shift in the maximum light absorption can be explained by the different sugar groups connected to the flavonoid’s skeleton, which can be distinguished using the UPLC-ESI-MS method (Supplementary Materials, Table S1, Figures S1–S3). The obtained results confirm previous studies by other authors [38,40].

The presented studies interpreted the effect on cell and lipid membranes only of a well-identified and described fraction of low-molecular polyphenols constituting part of the tested blueberry extracts. However, anthocyanins and phenolic acids, due to their chemical structure and small size, can interact to a greater extent with the cell membrane, both with the lipid phase, as shown by studies on RBCL membranes, and with the protein-lipid membrane of erythrocytes. In addition, anthocyanidins can electrostatically bind to the hydrophilic part of the erythrocyte membrane due to the large number of hydroxyl groups in their structure. Thus, they can protect membrane lipids from oxidation by scavenging and neutralizing free radicals from the environment [41].

In the second stage of the study, the antioxidant activity of HB, LB and WB extracts was examined in relation to the erythrocyte membranes. The antioxidant activity of the extracts was determined using fluorescent spectroscopy and applying two oxidation-inducing physicochemical agents: UVC radiation and the compound of AAPH. AAPH is a water-soluble small molecule often employed in studying lipid peroxidation and for the characterization of antioxidants. The thermal decomposition of an AAPH molecule generates two carbon-centred alkyl radical molecules, which then react with oxygen to produce peroxyl radical and alkoxyl radical molecules. The thermal decomposition rate of AAPH to form radical species averaged 2.1 × 10^−6^ s^−1^ and did not vary significantly with pH [53]. The main mechanism of free radical formation is the process of hydrolysis of the AAPH compound, so the free radicals are formed in the aqueous environment around the membrane.

Our investigations of antioxidant activity showed that the compounds contained in the extracts, in varying degrees, protect the erythrocyte membrane from oxidation induced by AAPH. The antioxidant potential of HB and LB extracts is comparable to the potential determined for Trolox^®^, a substance treated as a model antioxidant [51,52,55]. The authors’ previous studies have shown that chlorogenic acid and anthocyanidins: delphinidin-3-O-galactoside, cyanidin-3-O-glucoside and cyanidin-3-O-galactoside, which are the main components of the extracts, possessed comparable or better antioxidant activity to this determined for Trolox^®^, against free radicals generated by AAPH [41,45]. Obtained results indicate that the extracts of HB and LB, where the predominant ingredient is chlorogenic acid, had higher than WB antioxidant activity in relation to the erythrocyte membranes. Based on our earlier studies on the activity of chlorogenic acid, it can therefore be assumed that chlorogenic acid, which exhibits Trolox^®^-like antioxidant activity, is probably mainly responsible for their antioxidant activity [41]. However, it should be emphasized that combinations of several phytochemicals can cause a change in both the final biological effects and bioavailability of each component. The mixture of phenolic compounds can improve or reduce the benefits conferred by individual bioactive compounds and may induce facilitation/competition for cellular absorption and transport [56]. Thus, in this case, phytochemical interaction in extracts elicits antagonistic effects, which could explain the lower antioxidant yield of HB and LB extracts than the single chlorogenic acid.

Lipids undergo photo-oxidation by ultraviolet (UV) light, especially the most harmful UVC radiation (200–280 nm). Light radiation, particularly UV light, transforms triplet oxygen into highly reactive singlet oxygen ^1^O_2_, significantly accelerating the oxidation of unsaturated and polyunsaturated fatty acids (PUFAs). Photosensibilized oxidation leads to the generation of hydrogen peroxides and secondary oxidation products that differ considerably in molecular weight, beginning from low-molecular-weight compounds such as esters, ethers, and aldehydes to oxidized triacylglycerols (TAG) and polymers [57].

In addition, irradiation of erythrocyte membranes by UV results first in the oxidation of membrane proteins, the products of which may additionally cause the oxidation of membrane lipids. Tryptophan in peptides has been reported to photosensitize adjacent amino acids in polypeptide chains by an intramolecular electron transfer mechanism and lipid oxidation [58,59].

In relation to UVC-induced membrane lipids oxidation, the WB and HB extracts showed almost two times higher activity than LB extracts. However, this efficacy was almost two times lower than the efficacy of Trolox^®^. In particular, the significant difference in the activity of WB and LB extracts, which contain mainly chlorogenic acid, confirms previous findings that the activity of the extracts is the result of interactions between the components they contain. Anthocyanidins contained in the extracts, such as cyanidin-3-O-glucoside and malvidin-3-O-glucoside, protect against UVC-induced free radicals, which are more effective or comparable to Trolox^®^. In contrast, delphinidin-3-glucoside was a significantly weaker antioxidant than Trolox^®^, as our previous research has shown [45,60].

Additionally, statistically significant correlations could not be demonstrated between the antioxidant activity of the extracts and the quantity of low molecular weight phenolic compounds that occur in them. The highest content had been identified in the lowbush blueberry (LB) extract from the fruit (about 44% of dry weight), and its mild anti-oxidation activity to membranes was the lowest among the examined extracts. The extracts of the WB fruit contained approximately 34% of these compounds by dry weight, but their antioxidant activity compared to the extracts from LB fruit was more than twice higher when UVC radiation was the inducer of oxidation and only slightly higher when oxidation was induced by AAPH. HB extract, at which phenolic acids and flavonoids constituted about 39%, showed comparable and slightly better antioxidant activity than the LB extract against AAPH. In the case of UVC oxidation, its activity was smaller than WB extract but much better than LB extract. The lack of correlation between the antioxidant activity and the content of polyphenols indicates that not only the low-molecular polyphenolic compounds identified in the extracts, but also other components of the extracts are responsible for scavenging free radicals.

The degree of protection of the membrane against the harmful effects of free radicals depends on the type of oxidation inducer used (AAPH or UVC) and the type and percentage of low molecular weight polyphenols in the extract. The protection exerted by all tested red blood cell membrane extracts against free radicals induced by the compound AAPH was better compared with the protection against UVC radiation induced by free radicals [58]. In relation to UVC-induced membrane lipids oxidation, the wild bilberries extract showed the highest activity among the tested extracts. However, this efficacy was almost two times lower than that of Trolox^®^. Fluorimetric studies, in which free radicals were induced by AAPH, have shown that effective antioxidants are the extracts of lowbush and highbush blueberry fruit, which activity against free radicals was comparable to the activity of Trolox^®^.

Additionally, no statistically significant correlations were found between the antioxidant activity of the extracts and the quantity of low molecular weight phenolic compounds that occur in them. The highest content had been identified in the lowbush blueberry (LB) extract from the fruit (about 44% of dry weight), and its mild anti-oxidation activity to membranes was the lowest among the examined extracts. The extracts of the WB fruit contained approximately 34% of these compounds by dry weight, but their antioxidant activity compared to the extracts from LB fruit was more than twice higher when UVC radiation was the inducer of oxidation and only slightly higher when oxidation was induced by AAPH. HB extract, in which phenolic acids and flavonoids constituted about 39%, showed comparable and slightly better antioxidant activity than the LB extract against AAPH. In the case of UVC oxidation, its activity was smaller than WB extract but much better than LB extract. The lack of correlation between the antioxidant activity and the content of polyphenols indicates that not only the low-molecular polyphenolic compounds identified in the extracts, but also other components of the extracts are responsible for scavenging free radicals [61,62].

The studies have shown that the degree of protection of membrane lipids against the harmful effects of free radicals depends on the type of oxidation inducer used (AAPH or UVC), the type and percentage of low molecular weight polyphenols in the extract and the mutual interaction of them. The protection exerted by all tested extracts of the red blood cell membrane against free radicals induced by the AAPH compound was better compared with the protection against UVC radiation induced by the free radicals. Both inducers generate different types of free radicals, which are formed at different sites of the lipid bilayer. Thus, to better understand the mechanism of their antioxidant action, it is necessary to determine their location in the lipid bilayer. Research has therefore been carried out on the impact of the extracts on the physical properties of lipid model membranes and red blood cell membranes. Based on the effects of their interaction with membranes, the location of polyphenolic compounds in the membrane was determined. Biophysical studies were carried out on different membrane models, which facilitated interpreting the results on the effect of polyphenolic compounds and extracts on the biological membrane and lipid phase. The study of biological activity was carried out in relation to erythrocytes, isolated erythrocyte membranes (the ghosts) and lipid membranes. The interaction of the extracts with the membranes was determined based on physical changes incurred in the membranes, using the following techniques: optical and electron microscopic, UV–VIS and fluorimetric spectroscopy. The hemolytic and microscopic research was carried out on erythrocytes. The fluorimetric method was conducted on different models of biological membranes: erythrocytes and lipids membranes. The effects of polyphenolic compounds contained in the extracts on the membranes were determined based on induced hemolysis and changes in shape and osmotic resistance of erythrocytes, degree of order of the polar heads of erythrocyte lipids, and membrane fluidity in its hydrophobic area [63,64,65].

The hemolytic results showed that the polyphenolic extracts do not have a destructive effect on the membrane of red blood cells because they did not induce hemolysis in a wide range of the concentrations of tested extracts. This means that they can be safely used without risk of negative effects on the biological membrane, i.e., adverse effects in relation to membrane structures. This lack of hemolytic activity is confirmed by our previous studies, which showed that cyanidins and chlorogenic acid, the main components of the extracts, do not cause leakage of hemoglobin from the cells even at a very high concentration of 100 µM [66,67,68].

The test results showed that the extracts could not modify the osmotic resistance of red blood cells and the chlorogenic acid contained in the extracts [36]. The hemolytic and osmotic resistance studies have thus shown that the polyphenolic compounds contained in plant extracts do not work on membranes destructively. Therefore, it can be assumed that they do not penetrate deeply into the hydrophobic region of the cell membrane but only bind to its hydrophilic surface [66].

Microscopic examination showed that the substances change the discoidal shape of red blood cells and induce different forms of echinocytes, depending on the concentration. At higher concentrations of the extracts (0.1 mg/mL), we observed an increased percentage of echinocytes in the cell population, characterized by positive morphological indexes, i.e., disco-echinocytes (+1) and echinocytes (+2). Quantitative microscopic studies with a light microscope and an electron microscope showed that in the presence of extracts, the erythrocytes assume mainly various forms of echinocytes, indicating their presence in the outer lipid monolayer of the biological membrane. Furthermore, echinocytes formation is mainly induced by the extracts’ low molecular weight polyphenolic compounds, which exhibit this type of activity, i.e., anthocyanidins and chlorogenic acid [36,64]. The formation of echinocytes under the influence of different phenolic compounds was also observed by other authors, e.g., for caffeic acid [69] and extract from the leaves of *Buddleja globosa* [70]. It should be emphasized that the formation of echinocytes does not indicate a destructive effect on the membrane of the compounds causing their induction because these shapes are physiologically occurring in the blood, and their formation doesn’t break membrane integrity.

The results of the shape alteration of the erythrocytes showed the possibility of binding them to the erythrocyte membranes. Subsequent studies conducted with fluorescence steady-state spectroscopy at a constant temperature of 37 °C had to specify and locate the changes induced by polyphenolic substances. Studies were made for two membrane models: the simple model of liposomes formed from a mixture of various lipids and the lipid-protein erythrocyte membrane. They allowed the evaluation of the impact of polyphenols on the physical properties of the hydrophilic and hydrophobic parts of the membrane of erythrocytes and liposomes. The membranes of erythrocytes and liposomes differ in packing arrangement and lipid dynamics. The erythrocyte membrane, in relation to the lipid membrane, is more ordered and has a higher density of lipid packing and, consequently, less mobility due to, undoubtedly, the presence of proteins. It should also be noted that only the erythrocyte’s lipids are deliberately arranged asymmetrically in the bilayer, owing to the operation of adequate active and passive mechanisms. In contrast, the lipid distribution in monolayers in RBCL liposomes is coincidental [25].

Using two fluorescent probes: in conjunction with DPH and Laurdan, we examined the effect of extracts on the properties of the hydrophilic and hydrophobic regions of membranes. Modification of both these parts affects the intensity of fluorescence emitted by probes located in the respective areas and, consequently, the calculated values of fluorescence anisotropy (A) or generalized polarization (GP), respectively [66].

Studies of membrane fluidity have shown that polyphenolic extracts from the fruit of highbush, lowbush, and wild blueberries do not result in a significant change in the fluorescence anisotropy of the DPH probe and, therefore, do not alter substantially fluidity of two used models of the membrane in the hydrocarbon chains region. These results allow us to assume that polyphenolics bind to the surface of the membranes, interacting with them electrostatically or by hydrogen bonding. Therefore, we conclude that they do not penetrate the hydrophobic area of the membrane [60]. The slight decrease in the anisotropy of the erythrocyte ghosts could be an indirect result of the possible interaction of the components of the extracts, especially glycosylated polyphenols with glycocalyx and glycoproteins on the surface of the membrane of the erythrocyte (Table 2).

Changes in the hydrophilic part of membranes under the action of the tested substances are specified based on the generalized polarization (GP) of the Laurdan probe, that chromophore is incorporated at the glycerol level. The order parameter of the hydrophilic part, i.e., generalized polarization (GP), depends on the polarity of the chromophore environment. The decrease in the GP value meant that modifiacter caused an increase in the disorder of the hydrophilic area of the lipid bilayer, in particular, the changes in the lipid’s polar head orientation.

The presence of HB, LB and WB extracts causes a concentration-dependent decrease in the GP of the Laurdan probe. This result indicates that these extracts alter the arrangement of the hydrophilic region of the erythrocyte membrane, possibly by penetrating this region or being absorbed into the membrane surface.

The changes induced by extracts in the hydrophilic part of the membrane were significant only for the erythrocyte membrane. This effect may be related to the possible interactions of the extract components, particularly the sugar groups of polyphenols with the surface of the erythrocyte membrane and their glycocalyx, and the presence of integral and surface proteins in the membrane. The possibility of creating domains and lipid rafts using polyphenolic compounds is indicated by the authors of the work Tarahovsky et al., Koren et al. or Roy et al. [67,68,71]. More than 90% of the polyphenolic compounds identified in the WB extract are sugar compounds so that they can stabilize on the surface of the membrane. All experimental results indicate that polyphenolic compounds contained in the extracts are located in the hydrophilic region of the membrane and/or at the membrane surface. This location explains their high ability to protect membrane lipids against the free radicals induced by the AAPH compound and lower protection against UVC-induced lipid oxidation. The components of the extracts form a barrier on the membrane surface, preventing the diffusion of free radicals into its interior and effectively scavenging and neutralizing free radicals formed in the aqueous environment surrounding the membrane. Free radicals generated under the influence of UVC radiation are not only formed in the aqueous medium, as in the case of the AAPH compound but are also generated inside the membrane. The components of the extracts do not penetrate the hydrophobic part of the membrane, so they have limited access to the free radicals formed there. However, they can effectively scavenge and neutralize free radicals formed in the aqueous environment.

The small differences between the effects of polyphenolic compounds from three *Vaccinum* species with the biological and model membrane result from different qualitative compositions of the extracts. HB and LB extracts containing approximately 30% chlorogenic acid and 70% anthocyanins showed a similar capacity to modify the membrane surface. These extracts, through the presence of chlorogenic acid, shown in previous studies [36,69,72], cause changes on the surface of the membrane. Additionally, due to the small number of glycosylated anthocyanins, they could cause small changes in membrane hydration. On the other hand, WB extract containing more than 90% glycosylated anthocyanins modified the surface of the erythrocyte membrane in a slightly different way. Its constituents probably bind fewer water molecules on the surface of the membranes or create additional hydrogen bonds on the membrane surface with lipid molecules. Roy et al. [71] indicate strong dehydration of the liposome membrane and the formation of hydrogen bonds between the polar heads of the lipids and sugar groups. Changes in the hydration of the membrane surface determine the functioning of the membrane by affecting parameters such as hydration strength, dipole potential or maintaining a constant cell volume. Therefore, polyphenol compounds can be modulators of cell metabolic processes by acting intercellular signals by binding protein receptors on the surface of the membrane or by intercalating to the lipid bilayer of the membrane. Consequently, the indicated changes on the surface of the lipid membrane and erythrocyte membranes, and the same in its structure, are also significant at the cellular level in the case of HB, LB, and WB extracts, which may translate into their body protective properties. In protecting the erythrocyte’s membrane, obtained extracts can diminish the risk of blood and cardiovascular diseases and protect the cells’ membrane.

## 5. Conclusions

All experimental results show that polyphenol compounds contained in the highbush blueberry HB, lowbush blueberry LB and wild bilberry WB interact with the polar surface of the membrane and that they have practically no indirect influence on the fluidity of the hydrophobic region of the membranes. However, there are slight differences between interacting polyphenolic extracts with three ecotypes of *Vaccinum* L. blueberries with biological and model membranes, which may result in different quality content of the extract.

Based on the results obtained, it can be assumed that the protective effect of the extracts on biological membranes depends on the number of polyphenol molecules attached to the polar heads of lipids on the surface of the membrane, which pose a specific barrier. The molecular mechanism responsible for protecting biological membranes against the free radicals by these substances could be as follows. The polyphenolic constituents of the extract, when present in the medium and attached to the hydrophilic part of a membrane, reduce the concentration of the free radicals in the medium and constitute a barrier to those species which reach the surface of the membrane and also restrict diffusion of free radicals inside it.

In light of the results obtained, for prevention, it should be noted first of all the benefits for the body with a diet rich in fruits. The results of this research can be used to design new preparations or nanotechnological food ingredients involving polyphenols, which support the treatment and protection of the organism against diseases caused by oxidative stress.

Our results support using blueberry extracts in functional foods and food supplements to prevent chronic diseases associated with oxidative stress.

## Figures and Tables

**Table 1:** The content [%] and characterization of phenolic compounds of the extract of highbush (HB), lowbush (LB) and wild berries (WB) fruits using their spectral characteristics in HPLC–DAD (λ_max_) and negative and positive ions in UPLC–ESI–MS.

Compounds	Content [%] HB	Content [%] LB	Content [%] WB
Chlorogenic acid	11.26	11.82	1.19
Delphinidin-3-O-galactoside	2.65	7.97	5.72
Delphinidin-3-O-glucoside	2.75	4.16	6.12
Cyanidin-3-O-galactoside	6.30	2.61	2.60
Delphinidin-3-O-arabinoside	1.58	3.53	3.87
Cyanidin-3-O-glucoside	0.89	1.35	1.82
Petunidin-3-O-galactoside	0.64	1.73	0.62
Cyanidin-3-O-arabinoside	2.64	1.12	1.84
Petunidin-3-O-glucoside	0.87	2.02	1.35
Peonidin-3-O-galactoside	1.46	1.81	0.43
Petunidin-3-O-arabinoside	1.79	0.69	0.41
Peonidin-3-O-glucoside	0.92	0.12	1.87
Malvidin-3-O-galactoside	0.81	0.04	2.60
Peonidin-3-O-arabinoside	0.87	0.14	0.98
Malvidin-3-O-glucoside	0.21	0.04	0.85
Malvidin-3-O-arabinoside	0.73	0.21	0.44
Quercetin-3-O-arabinoside	0.15	0.05	-
Quercetin-3-O-rhamnoside	0.86	0.33	-
Quercetin-3-O-galactoside	0.29	3.50	0.72
Quercetin-3-O-glucoside	0.16	0.66	0.55
Total	37.83	43.90	33.98

**Table 2:** Values of IC_50_ for blueberry extracts, which inhibit erythrocyte membrane (ghost) oxidation by 50%, were determined with the fluorimetric method. The oxidation was induced with an AAPH inductor and UVC irradiation.

Extract/Inductor	Concentration IC_50_ [µg/mL] ± SD	
	AAPH	UVC
Wild bilberry (WB)	7.42 ± 0.37	21.19 ± 1.06
High blueberry (HB)	4.63 ± 0.23	26.34 ± 1.32
Low blueberry (LB)	4.96 ± 0.25	47.77 ± 2.38
Trolox^®^	3.90 ± 0.30	14.60 ± 1.30
chlorogenic acid *	0.90 ± 0.08	7.40 ± 0.50

* The results were published earlier by Bonarska-Kujawa et al. [53].

**Table 3:** Values of anisotropy (A) of the DPH probe in erythrocyte ghost and RBCL liposomes modified with different concentrations (5.0–25.0 µg/mL) of extracts from wild bilberry, high blueberry, and low blueberry at 37 °C. The experiment was carried out in at least three replicates. The results were presented as mean ± standard deviation. Statistically significant differences between modified and control membranes are marked: * α = 0.05.

Membrane	Erythrocyte Ghost	Liposomes from Erythrocyte’s Lipids
Concentrations [µg/mL]	Anisotropy(A) ± SD	
wild bilberry (WB)		
Control	0.24 ± 0.01	0.21 ± 0.01
5.0	0.24 ± 0.01	0.20 ± 0.01
7.5	0.25 ± 0.01	0.21 ± 0.01
10.0	0.23 ± 0.01	0.21 ± 0.01
25.0	0.23 ± 0.01	0.21 ± 0.01
high blueberry (HB)		
Control	0.24 ± 0.01	0.21 ± 0.01
5.0	0.24 ± 0.02	0.20 ± 0.01
7.5	0.22 ± 0.02	0.20 ± 0.01
10.0	0.23 ± 0.01	0.20 ± 0.01
25.0	0.22 ± 0.01 *	0.20 ± 0.01
low blueberry (LB)		
Control	0.24 ± 0.01	0.21 ± 0.01
5.0	0.24 ± 0.01	0.20 ± 0.01
7.5	0.24 ± 0.02	0.20 ± 0.01
10.0	0.22 ± 0.01	0.21 ± 0.01
25.0	0.22 ± 0.01 *	0.21 ± 0.01

**Table 4:** Values of generalized polarization (GP) of the Laurdan probe for erythrocytes membrane, RBCL liposomes modified with the different concentrations (5.0–25.0 µg/mL) of blueberry extracts from high blueberry (HB), low blueberry (LB) and wild bilberry (WB) at 37 °C. The experiment was carried out in at least three replicates. The results were presented as mean ± standard deviation. Statistically significant differences between modified and control membranes are marked: * α = 0.05.

Membrane	Erythrocyte Ghost	Liposomes from Erythrocyte’s Lipids
Concentrations [µg/mL]	Generalized Polarization (GP) ± SD	
wild bilberry (WB)		
Control	0.33 ± 0.01	0.27 ± 0.01
5.0	0.31 ± 0.02	0.27 ± 0.01
7.5	0.29 ± 0.01 *	0.27 ± 0.01
10.0	0.28 ± 0.02 *	0.26 ± 0.02
25.0	0.22 ± 0.03 *	0.27 ± 0.01
high blueberry (HB)		
Control	0.33 ± 0.01	0.27 ± 0.01
5.0	0.29 ± 0.03	0.27 ± 0.01
7.5	0.30 ± 0.01 *	0.27 ± 0.01
10.0	0.26 ± 0.02 *	0.27 ± 0.02
25.0	0.22 ± 0.02 *	0.26 ± 0.02
low blueberry (LB)		
Control	0.33 ± 0.01	0.27 ± 0.01
5.0	0.33 ± 0.01	0.27 ± 0.01
7.5	0.26 ± 0.01 *	0.28 ± 0.01
10.0	0.24 ± 0.03 *	0.27 ± 0.01
25.0	0.20 ± 0.03 *	0.25 ± 0.02

## Data Availability

The data presented in this study are available in this article.

## Supplementary Materials

The following supporting information can be downloaded at: https://www.mdpi.com/article/10.3390/membranes13060589/s1, Detailed description of UPLC–ESI–MS Analysis, -Table S1. Individual phenolic compounds identified by UPLC-PDA-MS/MS. Figure S1: Direct injection ESI-MS of polyphenols in the highbush blueberries (*Vaccinium corymbosum* L.) (HB) fruit extract. *m*/*z* value [M + H]^−^ positively charged molecular ions are 419.20—cyanidin-3-O-arabinoside; 433—peonidin-3-O-arabinoside; 435.26—delphinidin-3-O-arabinoside; 449.25—cyanidin-3-O-glucoside, cyanidin-3-O-galactoside and petunidin-3-O-arabinoside; 463.23—malvidin-3-O-arabinoside, peonidin-3-O-galactoside and peonidin-3-O-glucoside; 465—delphinidin-3-O-galactoside and delphinidin-3-O-glucoside; 479—petunidin-3-O-galactoside and petunidin-3-O-glucoside; 491.26—cyanidin-3-O-(6-O-acetyl)-glucoside; 493.28—malvidin-3-O-galactoside and malvidin-3-glucoside; 507—delphinidin-3-O-(6-O-acetyl) glucoside; 521.24—petunidin-3-O-(6-O-acetyl) glucoside; 535.23—malvidin-3-O-(6-O-acetyl) glucoside. Figure S2: Direct injection ESI-MS of polyphenols in the lowbush blueberries (*Vaccinium angustifolium* L.) (LB) fruit extract. *m*/*z* value [M + H]^−^ positively charged molecular ions are 419.20—cyanidin-3-O-arabinoside; 433—peonidin-3-O-arabinoside; 435.22—delphinidin-3-O-arabinoside; 449.20—cyanidin-3-O-glucoside, cyanidin-3-O-galactoside and petunidin-3-O-arabinoside; 463.20—malvidin-3-O-arabinoside, peonidin-3-O-galactoside and peonidin-3-O-glucoside; 465—delphinidin-3-O-galactoside and delphinidin-3-O-glucoside; 479—petunidin-3-O-galactoside and petunidin-3-O-glucoside; 491.21—cyanidin-3-O-(6-O-acetyl) glucoside; 493.21—malvidin-3-O-galactoside and malvidin-3-glucoside; 507—delphinidin-3-O-(6-O-acetyl) glucoside; 521.24—petunidin-3-O-(6-O-acetyl) glucoside; 535.21—malvidin-3-O-(6-O-acetyl) glucoside. Figure S3: Direct injection ESI-MS of polyphenols in the wild blueberries (*Vaccinium myrtillus* L.) (WB) fruit extract. *m*/*z* value [M + H]^−^ positively charged molecular ion are: 419.20—cyanidin-3-O-arabinoside; 433—peonidin-3-O-arabinoside; 435.19—delphinidin-3-O-arabinoside; 449.24—cyanidin-3-O-glucoside, cyanidin-3-O-galactoside and petunidin-3-O-arabinoside; 463.22—malvidin-3-O-arabinoside, peonidin-3-O-galactoside and peonidin-3-O-glucoside; 465—delphinidin-3-O-galactoside and delphinidin-3-O-glucoside; 479.26—petunidin-3-O-galactoside and petunidin-3-O-glucoside; 493.24—malvidin-3-O-galactoside and malvidin-3-glucoside.
