# Supplementary material for: Analysis of the Polyphenolic Composition of Vaccinium L. Extracts and Their Protective Effect on Red Blood Cell Membranes
Source: Membranes (Basel). 2023 Jun 7;13(6):589. doi: 10.3390/membranes13060589 (PMC10303154; doi:10.3390/membranes13060589)
Supplement: Supplementary file 1 [file membranes-13-00589-s001.zip › membranes-2420994-supplementary.pdf]

membranes

Supplementary Materials

Analysis of the Polyphenolic Composition of *Vaccinium* L. Extracts and Their Protective Effect on Red Blood Cell Membranes

Teresa Kaźmierczak [1,*], Dorota Bonarska-Kujawa [2], Katarzyna Męczarska [2], Sylwia Cyboran-Mikołajczyk [2], Jan Oszmiański [3] and Ireneusz Kapusta [4]

1 Department of Biotechnology, University of Wrocław, Joliot-Curie 14a, 50-383 Wrocław, Poland
2 Department of Physics and Biophysics, Wrocław University of Environmental and Life Sciences, Norwida 25, 50-375 Wrocław, Poland
3 Department of Fruit, Vegetable and Plant Nutraceutical Technology, Wrocław University of Environmental and Life Sciences, Chełmońskiego 37, 51-630 Wrocław, Poland
4 Institute of Food Technology and Nutrition, University of Rzeszów, Zelwerowicza 4, 35-601 Rzeszów, Poland
* Correspondence: teresa.kazmierczak09@gmail.com

UPLC–ESI–MS Analysis

Determination of polyphenolic compounds was carried out using the ultra-performance liquid chromatography (UPLC) Waters ACQUITY system (Waters, Milford, MA, USA). The UPLC system was equipped with a binary pump manager, column manager, sample manager, photodiode array (PDA) detector and tandem quadrupole mass spectrometer (TQD) with an electrospray ionisation (ESI) source. Separation of polyphenols was performed using a 1.7 µm, 100 mm × 2.1 mm UPLC BEH RP C18 column (Waters, USA). For the anthocyanin investigation, the mobile phase consisted of 2% formic acid in water, *v*/*v* (solvent A) and 2% formic acid in 40% acetonitrile, *v*/*v* (solvent B). However, in the case of other polyphenolic compounds, water (solvent A) and 40% acetonitrile, *v*/*v* (solvent B) were used. The flow rate was kept constant at 0.35 mL/min for a total run time of 8 min. The system was run with the following gradient program: from 0 min 5% B, from 0 to 8 min linear to 100% B and from 8 to 9.5 min for washing and back to initial conditions. The injection volume of the samples was 5 µL, and the column was supported at 50 °C. The following TQD parameters were used: cone voltage of 30 V, capillary voltage of 3500 V, source and desolvation temperature 120 °C and 350 °C, respectively, and desolvation gas flow rate of 800 L/h. Characterisation of the individual polyphenolic compounds was performed on the basis of the retention time, mass-to-charge ratio, fragment ions and comparison of data obtained with commercial standards and literature findings. Obtained data were processed in Waters MassLynx v.4.1 software (Waters, USA). The method was validated for parameters such as linearity, accuracy (relative error, RE), limit of detection (LOD), limit of quantification (LOQ), and precision (relative standard deviation, RSD). Quantification was determined by injection of solutions of known concentrations ranging from 0.05 to 5 mg ($R^2 \leq 0.999$) of the following phenolic compounds as standards: Chlorogenic acid, Delphinidin 3-O-glucoside, Cyanidin 3-O-glucoside, Malvidin 3-O-glucoside, Petunidin 3-O-glucoside, Peonidin 3-O-glucoside, Quercetin 3-O-rutinoside, (Extrasynthese, Genay Cedex, France). Stock standard solutions of the seven polyphenols were prepared with methanol. Six calibrators the peak area ratio of the polyphenol versus the nominal concentration. The regression equation was obtained by weighted (1/c2) least-squares linear regression. The LOD was determined as a signal-to-noise ratio (S/N) of 3:1, and the LOQ was determined as a S/N of >10. An acceptable RE within ±20% and the intra- and inter-day variations were determined using relative standard deviation (RSD) values were <3.5% for all the analyzed compounds.

Table 1. Individual phenolic compounds identified by UPLC-PDA-MS/MS.

Compound	Rt	λ_{max}	[M-H] m/z	
	min	nm	MS	MS/MS
Chlorogenic acid	2.21	288sh, 324	353^-	191
Delphinidin 3-O-galactoside	2.38	279, 523	465^+	301
Delphinidin 3-O-glucoside	2.67	259, 522	465^+	301
Cyanidin 3-O-galactoside	2.72	279, 515	449^+	287
Delphinidin 3-O-arabinoside	2.74	279, 523	435^+	303
Cyanidin 3-O-glucoside	2.92	279, 512	449^+	287
Petunidin 3-O-galactoside	3.31	277, 526	479^+	317
Cyanidin 3-O-arabinoside	3.43	279, 515	419^+	287
Petunidin 3-O-glucoside	3.53	278, 526	479^+	317
Peonidin 3-O-galactoside	3.85	278, 514	463^+	301
Petunidin 3-O-arabinoside	4.28	277, 523	449^+	317
Peonidin 3-O-glucoside	4.47	279, 521	463^+	301
Malvidin 3-O-galactoside	4.62	278, 530	493^+	331
Peonidin 3-O-arabinoside	4.68	279, 515	433^+	301
Malvidin 3-O-glucosides	4.73	279, 529	493^+	331
Malvidin 3-O-arabinoside	4.86	279, 528	463^+	317
Quercetin 3-O-arabinoside	4.93	255, 355	433^-	301
Quercetin 3-O-rhamnoside	5.00	255, 354	447^-	301
Quercetin 3-O-galactoside	5.23	255, 355	463^-	301
Quercetin 3-O-glucoside	5.35	255, 355	463^-	301

UPLC−ESI−MS spectra

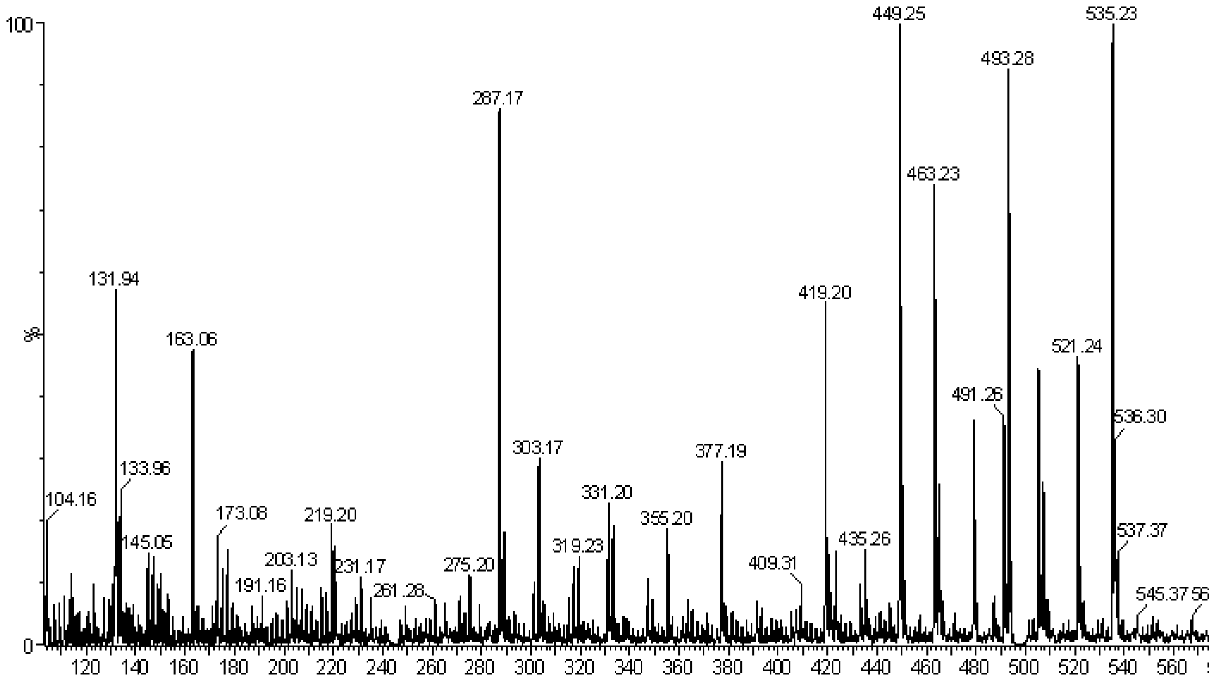

Figure S1. Direct injection ESI-MS of polyphenols in the highbush blueberries (*Vaccinium corymbosum* L.) (HB) fruit extract. m/z value $[M + H]^-$ positively charged molecular ion are: 419.20 — cyanidin-3-O-arabinoside; 433 — peonidin-3-O-arabinoside; 435.26 — delphinidin-3-O-arabinoside; 449.25 — cyanidin-3-O-glucoside, cyanidin-3-O-galactoside and petunidin-3-O-arabinoside; 463.23 — malvidin-3-O-arabinoside, peonidin-3-O-galactoside and peonidin-3-O-glucoside; 465 — delphinidin-3-O-galactoside and delphinidin-3-O-glucoside; 479 — petunidin-3-O-galactoside and petunidin-3-O-glucoside; 491.26 — cyanidin-3-O-(6-O-acetyl)-glucoside; 493.28 — malvidin-3-O-galactoside and malvidin-3-glucoside; 507 — delphinidin-3-O-(6-O-acetyl) glucoside; 521.24 — petunidin-3-O-(6-O-acetyl) glucoside; 535.23 — malvidin-3-O-(6-O-acetyl) glucoside.

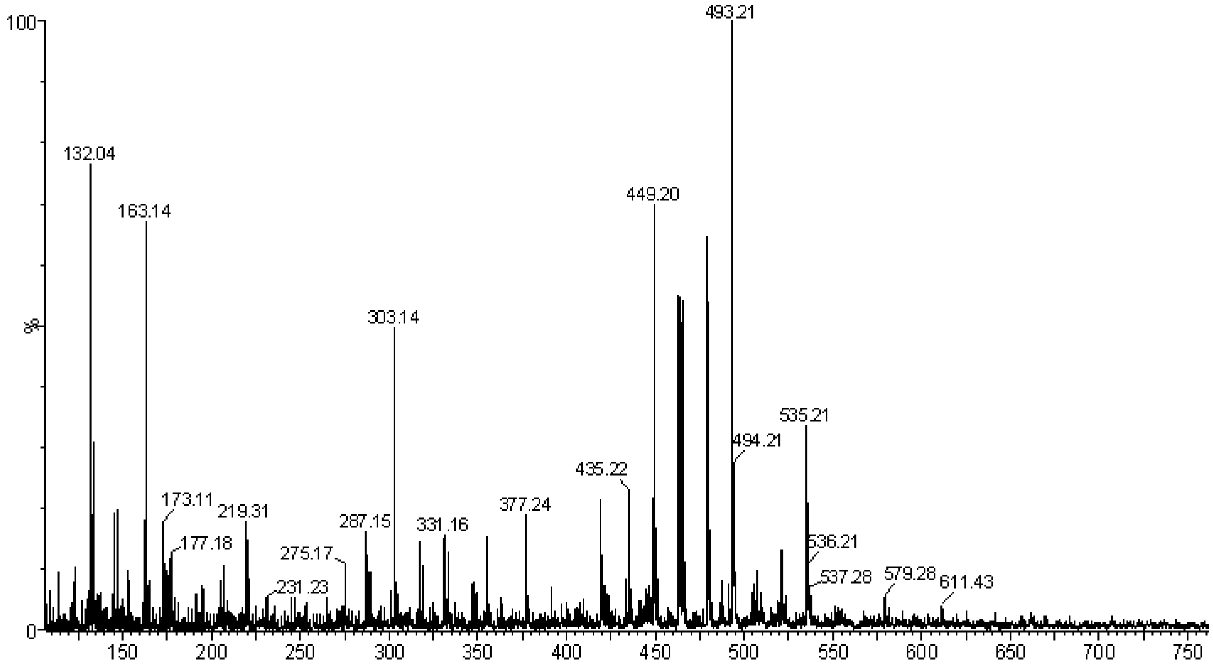

Figure S2. Direct injection ESI-MS of polyphenols in the lowbush blueberries (*Vaccinium angustifolium* L.) (LB) fruit extract. m/z value [M + H]$^{-}$ positively charged molecular ion are: 419.20 — cyanidin-3-O-arabinoside; 433 — peonidin-3-O-arabinoside; 435.22 — delphinidin-3-O-arabinoside; 449.20 — cyanidin-3-O-glucoside, cyanidin-3-O-galactoside and petunidin-3-O-arabinoside; 463.20 — malvidin-3-O-arabinoside, peonidin-3-O-galactoside and peonidin-3-O-glucoside; 465 — delphinidin-3-O-galactoside and delphinidin-3-O-glucoside; 479 — petunidin-3-O-galactoside and petunidin-3-O-glucoside; 491.21 — cyanidin-3-O-(6-O-acetyl) glucoside; 493.21 — malvidin-3-O-galactoside and malvidin-3-glucoside; 507 — delphinidin-3-O-(6-O-acetyl) glucoside; 521.24 — petunidin-3-O-(6-O-acetyl) glucoside; 535.21 — malvidin-3-O-(6-O-acetyl) glucoside.

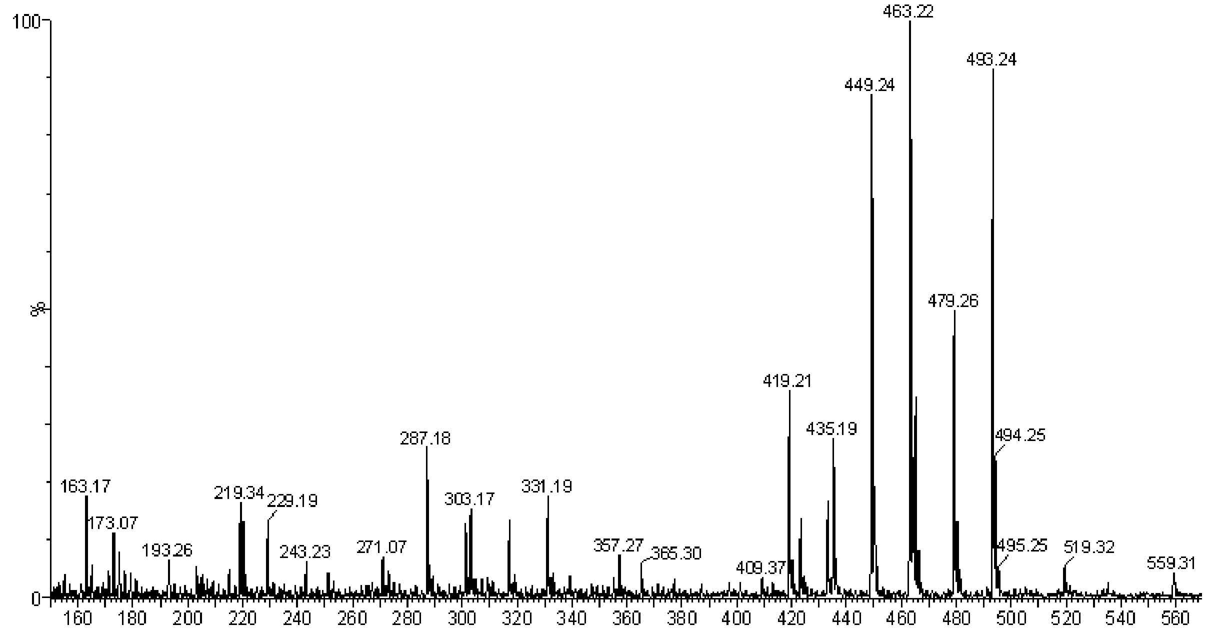

Figure S3. Direct injection ESI-MS of polyphenols in the wild blueberries (*Vaccinium myrtillus* L.) (WB) fruit extract. m/z value [M + H]$^{-}$ positively charged molecular ion are: 419.20 — cyanidin-3-O-arabinoside; 433 — peonidin-3-O-arabinoside; 435.19 — delphinidin-3-O-arabinoside; 449.24 — cyanidin-3-O-glucoside, cyanidin-3-O-galactoside and petunidin-3-O-arabinoside; 463.22 — malvidin-3-O-arabinoside, peonidin-3-O-galactoside and peonidin-3-O-glucoside; 465 — delphinidin-3-O-galactoside and delphinidin-3-O-glucoside; 479.26 — petunidin-3-O-galactoside and petunidin-3-O-glucoside; 493.24 — malvidin-3-O-galactoside and malvidin-3-glucoside.
